# Does Er,Cr:YSGG reduce the microleakage of restorations when used for cavity preparation? A systematic review and meta-analysis

**DOI:** 10.1186/s12903-020-01252-x

**Published:** 2020-10-06

**Authors:** Yali Zhang, Wenfei Chen, Jinrui Zhang, Yanhui Li

**Affiliations:** 1 Department of Stomatology, The First People’s Hospital of Qujing, Qujing, 655000 China; 2 Department of Stomatology, The Second People’s Hospital of Qujing, Qujing, 655000 China; 3 Department of Ophthalmology and Otorhinolaryngology, Zhanyi People’s Hospital of Qujing, Qujing, 655000 China

**Keywords:** Bur, Cavity preparation, Er,Cr:YSGG, Laser, Microleakage

## Abstract

**Background:**

As the member of erbium laser family, Erbium, Chromium: Yttrium Scandium Gallium Garnet (Er,Cr:YSGG) has obtained the approval for caries removal and cavity preparation by Food and Drug Administration (FDA). However, there is still controversy over the beneficial effects of Er,Cr:YSGG preparations on microleakage. The present study is the first systematic review and meta-analysis to compare the microleakage of cavities prepared by Er,Cr:YSGG lasers with that by traditional burs. In addition, the effect of acid etching on the adhesive potential of self-etch and etch-and-rinse adhesives was assessed after laser preparation.

**Methods:**

An electronic search was performed in Pubmed, EBSCO, Embase, and the Cochrane Controlled Register of Trials (CENTRAL).

**Results:**

Totally, 357 articles were identified. Finally, 13 met the inclusion criteria, of which 11 were selected for meta-analysis. All the included studies exhibited a moderate risk of bias. Based on the meta-analysis, no significant difference was observed between the Er,Cr:YSGG and traditional bur groups in terms of the incidence of microleakage. Self-etch adhesives, in combination with prior acid etching, showed less microleakage than those without acid etching in the laser-prepared cavities.

**Conclusions:**

Current studies do not support the beneficial effects of Er,Cr:YSGG preparations on microleakage. Additional acid etching with self-etching adhesives is recommended after Er,Cr:YSGG preparations. Further high-quality studies are needed to draw a convincing conclusion in the future.

## Background

Many new tools and materials have been developed owing to the popularity of minimally invasive dentistry. In the past 15 years, laser technology has attracted attention in modern dentistry for its various advantages [1, 2]. The member of erbium laser family, Erbium, Chromium: Yttrium Scandium Gallium Garnet (Er,Cr:YSGG) has gained the approval for caries removal and cavity preparation by Food and Drug Administration(FDA) [3]. Compared with traditional burs, Er,Cr:YSGG laser does not contact the tooth directly and has less vibration, noise, pressure, and thermal damage during cavity preparation [4]. Moreover, previous studies have reported a significant alteration in surface topography of the cavity after laser preparation, which might improve adhesion and the restorative procedure [5, 6]. Several researchers have measured the microleakage of cavities prepared by lasers and reported favorable results [7–9]. However, this conclusion is controversial because some studies have reported opposite results [10, 11]. Additionally, some researchers have recommended the use of acid etching in combination with self-etch and etch-and-rinse adhesives following laser preparation [9, 12]. To date, no systematic reviews have assessed the effect of Er,Cr:YSGG preparations on microleakage. Thus, this pioneering review was undertaken to assess: 1) the microleakage of cavities prepared by Er,Cr:YSGG lasers in comparison with that by traditional burs; 2) the effect of acid etching on the adhesive potential of self-etch and etch-and-rinse adhesives after laser preparation.

## Methods

This systematic review and meta-analysis was performed according to the Preferred Reporting Items for Systematic Reviews and Meta-Analyses (PRISMA) guidelines [13].

### Focused question

The focused question according to the Participants, Interventions, Control and Outcomes (PICO) principle was: ‘During cavity preparation, does Er,Cr:YSGG laser result in less microleakage of the restoration in comparison to traditional burs?’ and ‘Does the application of prior acid etching increase the adhesive potential of self-etching and etch-and-rinse adhesives in laser-prepared cavities?’

### Search strategy

A literature search was conducted in Pubmed, EBSCO, Embase, and the Cochrane Controlled Register of Trials (CENTRAL) up to July 2019. Appropriate search algorithms were developed for each database, using the following Boolean phrases: (laser) AND (microleakage OR leakages, dental OR dental leakage OR leakage, dental) AND (cavity preparation OR preparation, dental cavity OR preparations, dental cavity OR cavity preparation, dental OR dental cavity preparations). The search had no restriction in the publication language.

Two blinded, independent investigators screened the titles and abstracts from the electronic searches to find potentially eligible studies. The full texts of all the seemingly eligible studies were obtained and further evaluated in detail to make sure they really met all the selective criteria. To avoid missing eligible studies, the reference lists of all the selected full-text studies were also screened. The agreement between the reviewers was calculated in the selection procedure by Cohen’s kappa statistics [14], which yielded a value of 0.93. Any discrepancies were resolved by discussion until a consensus was reached.

### Inclusion criteria

The investigators selected the included studies based on the following criteria:

1. Randomized controlled trials (RCTs) or quasi-RCTs.

2. Comparison of the microleakage of cavities prepared by Er,Cr:YSGG laser versus cavities prepared by traditional burs.

3. Comparison of the microleakage of cavities prepared by laser with additional acid etching with that by laser alone.

4. Use of human teeth.

5. Drop-out rate: < 20%.

### Exclusion criteria

Case reports, review papers, letters to the editor, monographs, conference abstracts, and animal studies were excluded.

### Data collection and analysis

Two reviewers independently extracted and managed data on the characteristics of the included studies as follows: year of publication, the country of origin, study design, number of teeth, restorative materials and adhesive systems used, cavity type, cavity size, tooth type, groups, number of drop-outs, and microleakage test. The following parameters of Er,Cr:YSGG laser were also recorded: mode, manufacturer, tip, wavelength, pulse frequency, pulse duration, mean power, and method of application. Any difference was resolved by discussion. In cases in which research data were incomplete or missing, the authors were contacted to ask for further information.

Statistical analysis was performed using RevMan 5.3 software provided by the Cochrane Collaboration. Risk ratio (RR) was used, along with 95% confidence intervals (CIs), for dichotomous data, while the mean difference (MD) was used with 95% CIs for continuous data. In addition, the z-test was used. I^2^ test on the level of α = 0.10 was used to evaluate statistical heterogeneity. When there was significant statistical heterogeneity (I^2^ > 50%), a random-effect model was used to analyze the data. If I^2^ was ≤50%, a fixed-effect model was used. The statistical significance for the hypothesis testing was set at α < 0.05 (2-tailed z-tests). When the data could not be pooled in the meta-analysis, they were summarized qualitatively.

### Quality assessment

Two investigators independently assessed the quality of the target studies using Cochrane Collaboration’s tool for assessing the risk of bias. The assessment for each article included seven domains, and each domain was divided into three categories: low risk of bias, unclear risk of bias, and high risk of bias. The study was judged as low risk of bias if all the domains were deemed low risk, as moderate risk if one or more were considered as unknown risk, as high risk if all were deemed high risk. The agreement between the reviewers was assessed based on Cohen’s kappa statistics, assuming κ = 0.6 to be a favorable score. Any disagreement was resolved by discussion, and a third investigator was consulted if arbitration was required.

## Results

### Study characteristics

**Fig. 1:**
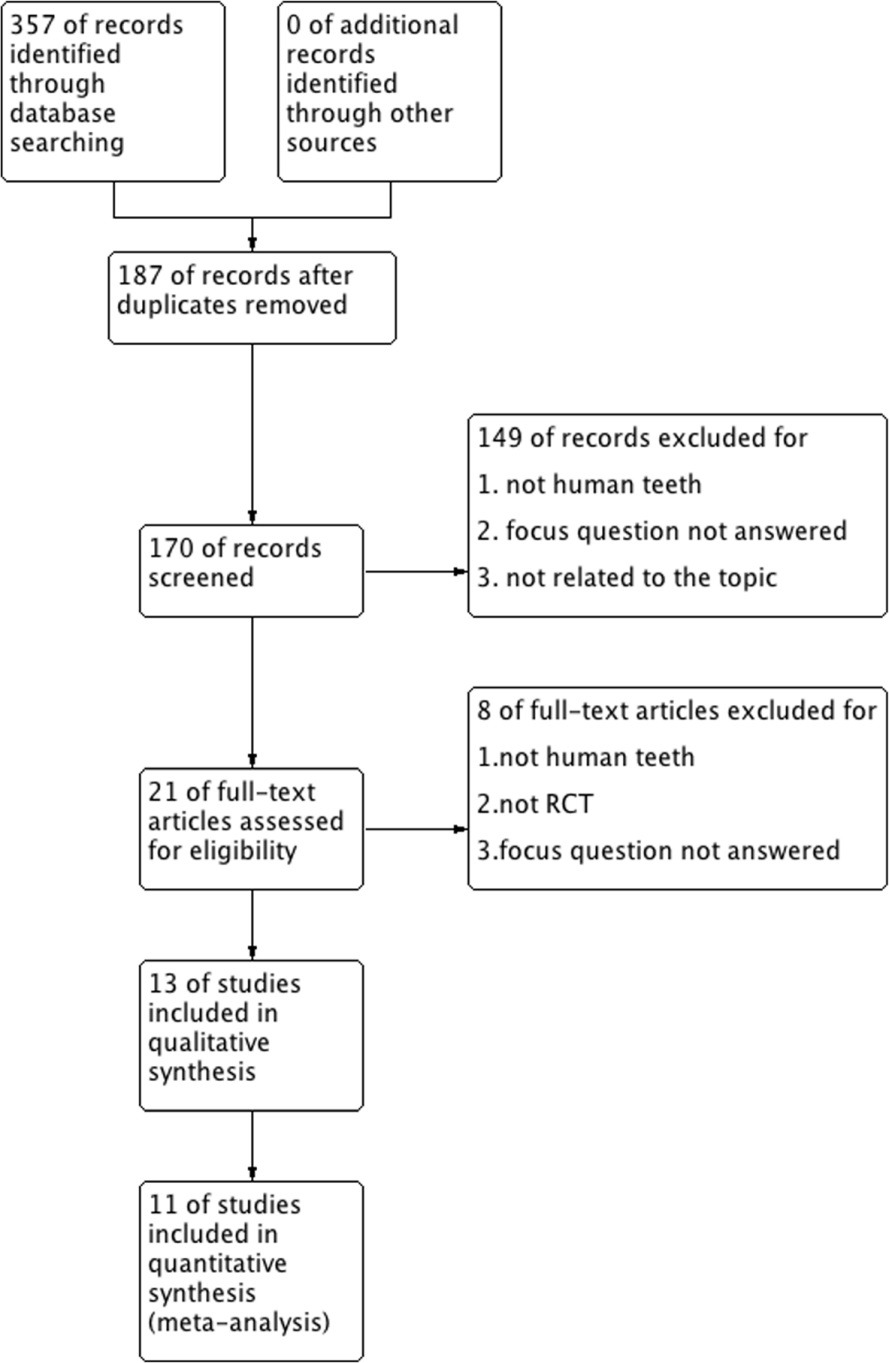
Flowchart depicting the study selection process

Totally, 357 articles were retrieved initially from the databases, which decreased to 170 after removing the duplicates (Fig. 1). After screening the titles and abstracts, the full texts of 21 articles were obtained for more detailed data. Finally, 13 studies were included, 11 of which were selected for meta-analysis [4–9, 12, 15–18].

Table 1 presents the characteristics of the studies included. The 13 studies were published from 2001 to 2018; five studies were conducted in Iran, two in Turkey, two in Brazil, and the remaining were conducted in Germany, Spain, Brazil, and India, respectively. Among them, five studies selected primary teeth as the experimental models, while the others selected premolars, third molars, or other posterior teeth. Class V cavities accounted for 10 out of 13 studies. Table 2 presents the parameters of the lasers used.

**Table 1:** Characteristics of the included studies

Study ID	Country	Study design	No. of teeth	Restoration material and adhesive system	Cavity type	Cavity size	Tooth type	Groups	No. of drop-out	Microleakage test
Malekafzali 2017 [4]	Iran	Parallel	30	G1–3:Flowable composite (Grandio flow, Voco, Germany)/Etch-and-rinse adhesive (Solobond M, Voco, Germany) + 35%PA	Class V	MD:3 mm OG:2 mm Depth:1.5 mm	Primary canine teeth	G1:Diamond bur G2:Er, YAG G3:Er, Cr:YSGG	None	Enamel margin: 0:No dye penetration 1:Dye penetration maximally extending to the dentinoenamel junction 2: Dye penetration passing the dentinoenamel junction but not reaching the axial wall 3:Dye penetration reaching the axial wall 4: Dye penetration reaching dental pulp Dentine margin: 0: No dye penetration 1: Dye penetration less than half the diatance to the axial wall 2: Dye penetration extending more than half the distance to the axial wall but not reaching it 3: Dye penetration to axial wall 4: Dye penetration reaching dental pulp
Ergin 2018 [6]	Turkey	Parallel	54	S1:Methacrylate-based microhybrid composite (Filtek P60, 3 M ESPE)/Etch-and-rinse adhesive (Adper Single Bond 2, 3 M ESPE) + 37%PA S2:Silorane(Filtek Silorane, 3 M ESPE)/Self-etch adhesive(Silorane System Adhesive, 3 M ESPE) S3:Nanohybrid methacrylate-based composite (Kalore, GC) + self-etch adhesive(G-Bond, GC)	Class II	BL:2.5 mm Depth:1.5 mm	Premolar	G1–3:Diamond bur G4–6: Er, Cr:YSGG+MD handpiece G7–9: Er, Cr:YSGG+Turbo handpiece	None	Enamel margin: 0: No dye penetration 1:Dye penetration observed only on enamel 2: Dye penetration observed beyond the dentino-enamel junction 3: Dye penetration observed up to the pulpal wall Dentine margin: 0: No dye penetration 1: Dye penetration observed up to half the cervical wall 2: Dye penetration observed more than half the cervical wall 3: Dye penetration observed up to the axial wall
Subramaniam 2016 [5]	India	Parallel	40	G1:Composite resin(Filtek 350 XT, 3 M ESPE)/Etch-and-rinse adhesive(Adper Single Bond 2, 3 M ESPE) + 35%PA G2: Composite resin(Filtek 350 XT, 3 M ESPE)/Etch-and-rinse adhesive(Adper Single Bond 2, 3 M ESPE)	Class III	Depth:0.5-1 mm Height:2 mm Width:2 mm	Primary upper and lower anterior teeth	G1: Diamond bur G2: Er, Cr:YSGG	None	0: No dye penetration 1:Dye penetration up to enamel 2:Dye penetration up to dentine 3: Dye penetration involving floor of cavity
Shafiei 2014 [12]	Iran	Parallel	56	G1/G3:Silorane composite(Filtek Silorane, 3 M ESPE)/Self-etch adhesive(Silorane Adhesive, 3 M ESPE) G2/G4:Silorane composite(Filtek Silorane, 3 M ESPE)/Self-etch adhesive(Silorane Adhesive, 3 M ESPE) + 35%PA	Class V	Width:2 mm Height:2 mm Depth:1 mm	Primary canine teeth	G1/2: Diamond bur G3/4: Er, Cr:YSGG	None	0:No dye penetration 1: Dye penetration along the cavity wall but less than one half the length 2: Dye penetration along the cavity wall but short of the axial wall 3: Dye penetration along the axial wall
Fattah 2013 [8]	Iran	Parallel	68	G1/G3:Composite resin(Filtek Z250 shade A2, 3 M ESPE)/Etch-and-rinse adhesive(Adper single Bond 2, 3 M ESPE) + 35%PA G2/G4: Composite resin(Filtek Z250 shade A2, 3 M ESPE)/Etch-and-rinse adhesive(Adper single Bond 2, 3 M ESPE)	Class V	Length:4 mm Width:3 mm Depth:1.5 mm	premolar	G1/2:Diamond bur G3/4:Er, Cr:YSGG	None	0:No dye penetration 1: Dye penetration up to one third of occlusal and/or gingival walls 2: Dye penetration up to two thirds of occlusal and/or gingival walls 3: Dye penetration of occlusal and/or gingival walls up to axio-occlusal and/or axio-gingival intersection 4: Dye penetration along axial wall
Rossi 2008 [19]	Brazil	Parallel	100	G1/3/5/7/9:Ketac Molar Easy Mix(CGIC, 3 M, St Paul, Minn) G2/4/6/8/10:Vitremer(RMGIC, 3 M)	Class V	Diameter:3 mm Depth:2 mm	Primary canine teeth	G1–2:Diamond bur G3–10: Er, Cr:YSGG	None	0:No dye penetration 1: Dye penetration up to one third of the cavity depth 2: Dye penetration up to two third of the cavity wall depth 3: Dye penetration to but not along the axial wall 4: Dye penetration up to and along axial wall
Yazici 2012 [15]	Turkey	Parallel	40	G1–4:Nanohybrid composite resin(Premise, Kerr, Orange, CA)/Etch-and-rinse adhesive(Adper Single Bond 2, 3 M ESPE) + 37%PA)/ Self-etch adhesive(AdheSE One, Ivoclar Vivadent, Schaan, Liechtenstein)	Class V	MD:3 mm OG:2 mm Depth:1.5 mm	Premolar	G1:Diamond bur G2:Carbide bur G3:Er, Cr:YSGG G4:CVD bur	None	0: No dye penetration 1: Partial dye penetration along the occlusal/gingival wall 2: Dye penetration along the occlusal/gingival wall but not including axial wall 3: dye penetration to and along the axial wall
Marotti 2010 [18]	Brazil	Parallel	100	G1/2:Composite resin(Z250, 3 M ESPE)/Self-etch adhesive(Single Bond, 3 M ESPE) + 37%PA G3/5/7/9: Composite resin(Z250, 3 M ESPE)/Self-etch adhesive(Single Bond, 3 M ESPE) + laser etching G4/6/8/10: Composite resin(Z250, 3 M ESPE)/Self-etch adhesive(Single Bond, 3 M ESPE) + laser etching+ 37%PA	Class V	Length:3 mm Width:3 mm Depth:2 mm	Third molar	G1: Diamond bur G2–10: Er, Cr:YSGG	None	0: No dye penetration 1: Dye penetration up to enamel 2: Dye penetration up to dentine 3: Dye penetration involving the pulpal floor of the cavity
Gutknecht 2001 [16]	Germany	Parallel	24	G1:Hybrid composite resin(Kerr)/Etch-and-rinse adhesive(Optibond, Kerr, Karlsruhe, Germany) + 37%PA G2:Hybrid composite resin(Kerr)/Etch-and-rinse adhesive(Optibond, Kerr, Karlsruhe, Germany) G3:Hybrid composite resin(Kerr)/Etch-and-rinse adhesive Optibond, Kerr, Karlsruhe, Germany) + 37%PA	Class II	NR	Third molar	G1: Diamond bur G2–3: Er, Cr:YSGG	None	0:No dye penetration 1:Penetration into the enamel part of the cavity wall 2: Penetration into the dentin part of the cavity wall 3: Penetration including the pulpal floor of the cavity
Shahabi 2008 [7]	Iran	Parallel	30	G1:Composite resin(Vivadent, Liechtenstein)/Etch-and-rinse adhesive(Excite, Vivadent, Liechtenstein) + 37%PA G2: Composite resin(Vivadent, Liechtenstein)/Etch-and-rinse adhesive(Excite, Vivadent, Liechtenstein) G3: Composite resin(Vivadent, Liechtenstein)/Etch-and-rinse adhesive(Excite, Vivadent, Liechtenstein) + 37%PA	Class V	Height:2 mm Width:4 mm Depth:2 mm	Permanent posterior teeth	G1: Diamond bur G2–3: Er, Cr:YSGG	None	0: No dye penetration 1: Dye penetration reaching the enamel or cementum 2: Dye penetration reaching the dentine 3: Dye penetration reaching the cavity floor
Shafiei 2015 [9]	Iran	Parallel	56	G1/3:Ketac N100(3 M ESPE,USA)/Self-etch adhesive(Ketac Nano primer, 3 M ESPE) G2/4: Ketac N100(3 M ESPE,USA)/Self-etch adhesive(Ketac Nano primer, 3 M ESPE) + 35%PA	Class V	Height:2 mm Width:2 mm Depth:0.5 mm	Primary canine	G1–2: Diamond bur G3–4: Er, Cr:YSGG	None	0:No dye penetration 1: Dye penetration along the cavity wall but less than one half the length 2: Dye penetration along the cavity wall but short of the axial wall 3: Dye penetration along the axial wall
Geraldo-Martins 2013 [20]	Brazil	Parallel	70	G1–14:Flowable composite(Palfque Estelite LV)/Self-etch adhesive(Clearfil SE Bond, Kuraray Medical Inc)		The removal of carious lesions determined the form of the cavity	Molars and premolars	G1:Bur G2–14: Er, Cr:YSGG(1,1.25,1.5,1.75,2,2.25,2.5,2.75,3,3.25,3.5,3.75,4 W)		The total length of the tooth/restoration interface was measured. Then the length of the interface infiltrated by dye was calculated. These data were used to calculate the infiltration index for each section as the percentage of the interface length showing infiltration
Trelles 2012 [17]	Spain	Parallel	30	G1–3:Composite resin(Clearfil Majesty)/Self-etch adhesive(Clearfil SE Bond, Kuraray Medical Inc)	Class V	Length:6 mm Width:4 mm Depth:2 mm	Third molar	G1:Bur G2: Er, Cr:YSGG(4 W) G3: Er, Cr:YSGG(1.5 W)	None	0:No dye penetration 1:Dye penetration less than one-third of the cavity wall 2: Dye penetration less than two-third of the cavity wall 3: Dye penetration more than two-third of the cavity wall without axial wall involvment 4: Dye penetration to the full extent of the cavity wall,reaching the axial wall od penetrating it

*NO* number*, MD* mesiodistal, *OG* occlusogingival, *BL* buccolingual, *NR* not reported, *G* group, *S* subgroup, *PA* phosphoric acid

**Table 2:** Laser parameters of the included studies

Study ID	Mode	Manufactures	Tip	Wavelength	Pulse frequency	Pulse duration	Mean power	Method of application
Malekafzali 2017 [4]	NR	WaterLase iplus, Biolase, USA	MZ8	2.78 μm	15 Hz	NR	3 W(60% air; 30% water)	NR
Ergin 2018 [6]	Uncontact	Biolase Millennium II, Biolase Technologies, San Clemente, CA	MG6 MX5	2.78 μm	20 Hz	140 μs	5 W(70% air; 60% water;)	The beam was aligned vertically to the target tissue at a distance of 1–1.5/3-5 mm and moved in a sweeping motion
Subramaniam 2016 [5]	Uncontact	Biolaseiplus Technology, USA	NR	2.78 μm	15 Hz	600–700 μs	Enamel:4 W (60% air; 60% water) Dentine:3 W(60% air; 30% water)	The laser was placed 8–10 mm away from the teeth
Shafiei 2014 [12]	Focus	Waterlase, Biolase, Irvine, CA	G4	2.78 μm	20 Hz	40–200 μs	Enamel:3 W(85% air; 85% water) Dentine:2 W (65% air; 55% water)	NR
Fattah 2013 [8]	Free-running pulse	Waterlase, Biolase Technology, San Clemente, CA	G6	2.78 μm	20 Hz	140 μs	3.5 W(65% air; 55% water)	The laser was placed 1–2 mm away from the teeth
Rossi 2008 [19]	NR	Millennium, Biolase Technology, San Clemente, CA	G6	2.78 μm	20 Hz	140–200 μs	Enamel:2.5/3 W(55% air; 65% water) Dentine:1/1.5 W(55% air; 65% water)	The laser was placed perpendicularly to the surface and 1 mm away from the teeth
Yazici 2012 [15]	Uncontact	Millennium II, Biolase Technology, Irvine, CA	G4	NR	20 Hz	140 μs	Enamel:5.25 W(90% air; 75% water) Dentine:3.5 W(65% air; 55% water)	The laser was placed 2–3 mm away from the teeth
Marotti 2010 [18]	NR	Waterlase, Biolase Technology, San Clemente, CA	NR	2.78 μm	20 Hz	140 μs	5 W(90% air; 70% water)	NR
Gutknecht 2001 [16]	NR	Millennium, Biolase Technology	NR	2.78 μm	20 Hz	140 μs	Enamel:6 W Dentine:5 W	The laser was placed 0.5 mm away from the teeth
Shahabi 2008 [7]	NR	Waterlase, Biolase, USA	NR	2.78 μm	20 Hz	140 μs	Enamel:5.5 W(95% air; 80% water) Dentine:3.5 W(75% air; 65% water)	The laser was placed 1.5–2 mm away from the teeth
Shafiei 2015 [9]	Focus	Waterlase, Biolase, Irvine, CA	G4	2.78 μm	20 Hz	140–200 μs	Enamel:3 W(85% air; 85% water) Dentine:2 W(65% air; 55% water)	NR
Geraldo-Martins 2013 [20]	Pulse	Waterlase, Biolase Technology, San Clemente, CA	NR	2.78 μm	20 Hz	140 μs	1,1.25,1.5,1.75,2,2.25, 2.5,2.75,3,3.25,3.5,3.75,4 W(55% air; 65% water)	NR
Trelles 2012 [17]	Pulse	Waterlase, Biolase Technology, San Clemente, CA	G4	2.78 μm	10-40 Hz	140–200 μs	High energy:4 W(50% air; 50% water) Low energy:1.5 W(30% air; 30% water)	The laser was placed perpendicularly to the surface and 1.5–1.7 mm away from the teeth

*NR* not reported, *CA* California, *USA* the United States

### Quality analysis

**Fig. 2:**
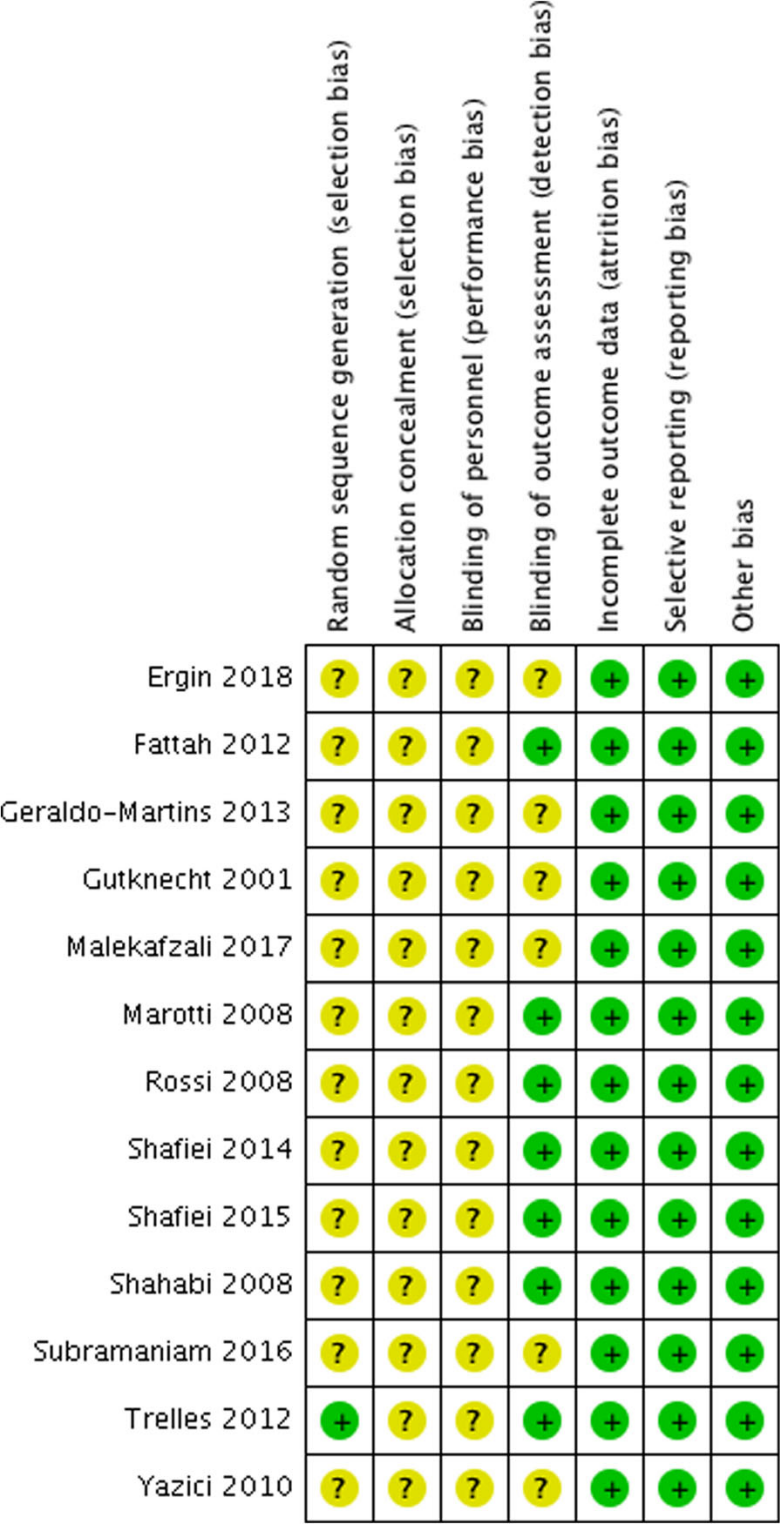
Risk of bias summary

As shown in Fig. 2, all the included studies were considered to have a moderate risk of bias (Fig. 2). Among them, only one study described the randomization methods clearly [17], and five studies referred to the blinding of outcome assessment [4–6, 15, 16]. All the studies had unclear information about ‘allocation concealment’ and ‘blinding of wpersonnel.’

### Primary outcomes: the effect of Er,Cr:YSGG preparations on microleakage

**Fig. 3:**
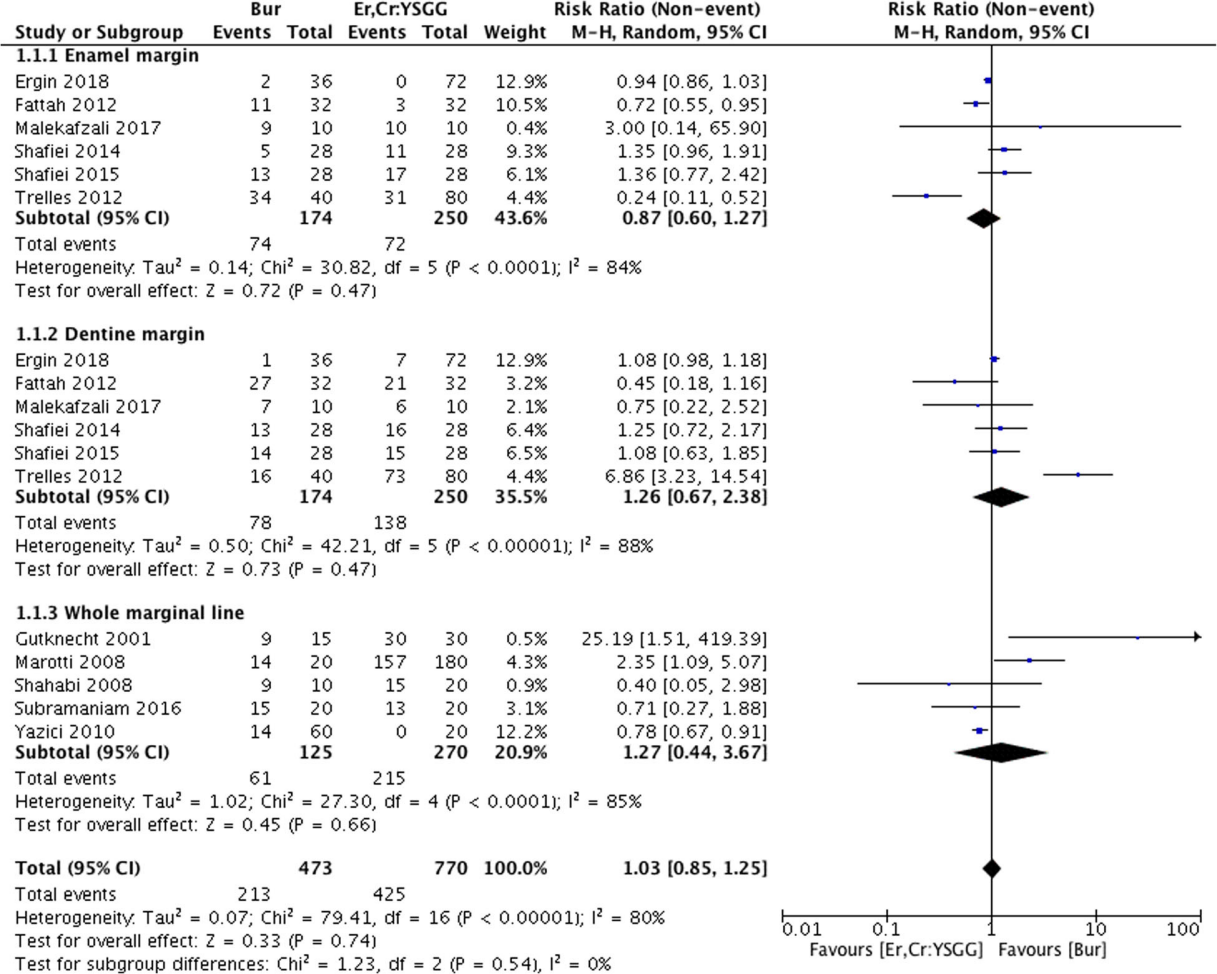
Comparison: Bur preparations versus Er,Cr:YSGG preparations, outcome: the incident rate of microleakage

Except for two studies that did not report specific microleakage scores or indexes [19], the remaining 11 studies were selected for meta-analysis (Fig. 3). Subgroup analysis was conducted based on different measuring positions (enamel margin, dentin margin, and the whole marginal line). Six studies compared the microleakage of cavities prepared by burs with that prepared by Er,Cr:YSGG lasers on enamel and dentin margins [4, 6, 8, 9, 12, 17], while the remaining five measured microleakage on all the marginal lines [5, 7, 15, 16, 18]. The results revealed significant heterogeneity among the studies (χ^2^ = 79.41, I^2^ = 80%, *P* < 0.00001). Meta-analysis with a random model indicated that the incidence of microleakage was a little higher in traditional bur groups both on the dentin and the whole marginal line, while it did not show any significant difference (RR = 1.03, 95% CI range: 0.85–1.25, *P* = 0.74), (dentin margin: RR = 1.26, 95% CI range: 0.67–2.38, *P* = 0.47; whole marginal line: RR = 1.27, 95% CI range: 0.44–3.67, *P* = 0.66). However, in the enamel margin subgroup, the results revealed an insignificant increase in microleakage in the laser group (RR = 0.87, 95% CI range: 0.60–1.27, P = 0.47). The study by Rossi et al., which was excluded from the meta-analysis, also reported no statistically significant difference between the bur and Er,Cr:YSGG laser groups on the whole marginal line [19]. However, Geraldo-Martins et al. reported that the Er,Cr:YSGG group had a higher microleakage index compared to the traditional bur group [20].

### Secondary outcomes: the effects of acid etching on microleakage in Er,Cr:YSGG-prepared cavities

**Fig. 4:**
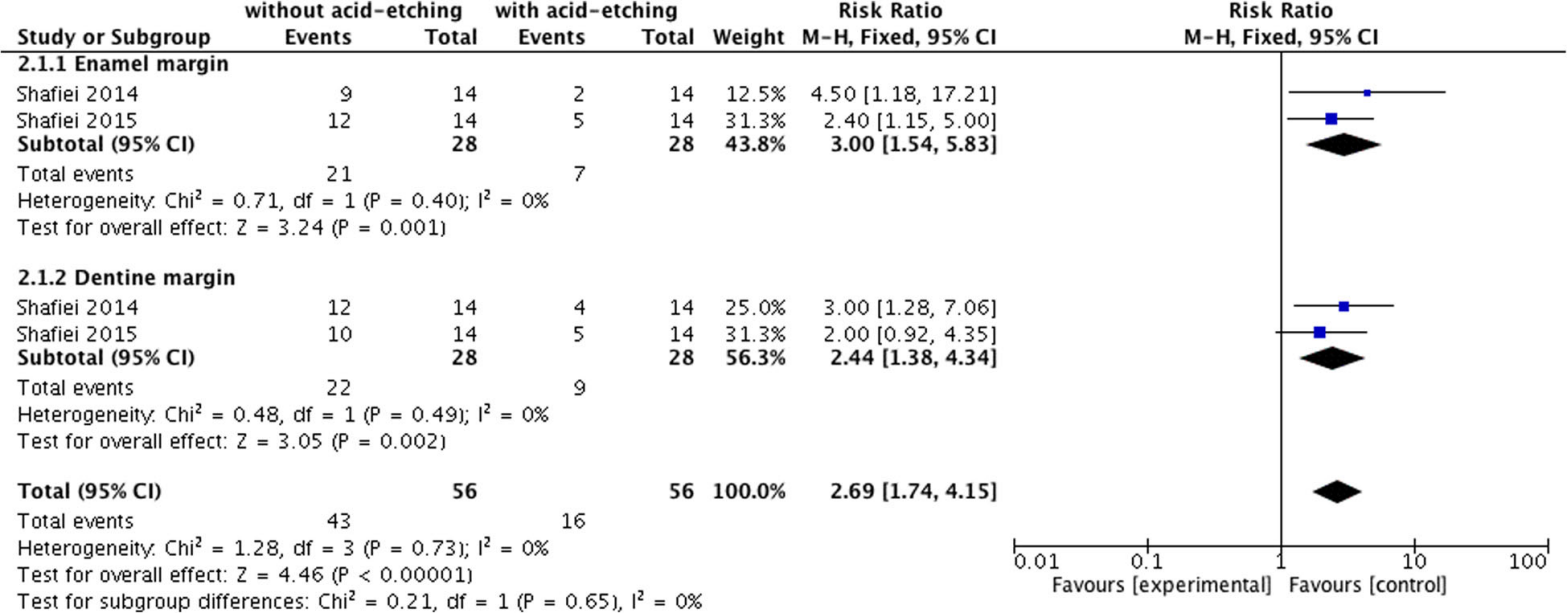
Comparison: Er,Cr:YSGG preparations without additional acid etching versus Er,Cr:YSGG preparations with acid etching (using self-etch adhesive systems), outcome: the incident rate of microleakage

**Fig. 5:**
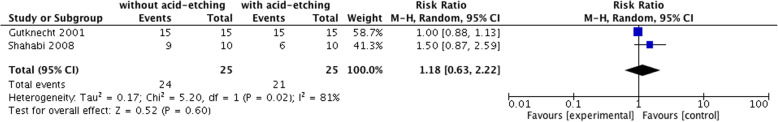
Comparison: Er,Cr:YSGG preparations without additional acid etching versus Er,Cr:YSGG preparations with acid etching (using etch-and-rinse adhesive systems), outcome: the incident rate of microleakage

Four out of 13 studies measured the effect of acid etching on the adhesive potential of self-etch and etch-and-rinse adhesives after laser preparation [7, 9, 12, 16]. Two studies used acid etching with self-etching adhesives and evaluated the microleakage value on enamel and dentin margins, respectively [9, 12], while the remaining two applied etch-and-rinse adhesives and measured the value on the whole marginal lines [7, 16]. It was reported that prior acid etching improved the adhesive potential of self-etching adhesives and decreased microleakage after laser preparations significantly (χ^2^ = 1.28, I^2^ = 0%, *P* = 0.73, RR = 2.69, 95% CI range: 1.74–4.15, *P* < 0.00001). The significant difference was detected both in the enamel and dentin margin subgroups (enamel margin: RR = 3.0, 95% CI range: 1.54–5.83, *P* = 0.001; dentin margin: RR = 2.44, 95% CI range: 1.38–4.34, *P* = 0.002) (Fig. 4). However, the difference was not significant for the etch-and-rinse adhesives (χ^2^ = 5.20, I^2^ = 81%, *P* = 0.02, RR = 1.18, 95% CI range: 0.63–2.22, *P* = 0.60) (Fig. 5).

## Discussion

### Quality of the studies

Figure 2 presents the quality of the included studies. All the studies were considered to have a moderate risk of bias. Unclear information about ‘random sequence generation,’ ‘blinding of personnel’ and ‘allocation concealment’ were the main risk factors. Although all the studies were conducted randomly, only one study mentioned the randomization method adopted in the experiment. Computer programs, random tables, or other randomization methods are necessary to balance the assignment to experimental and control groups. In addition, the importance of allocation concealment and blinding techniques must be emphasized. Improper blinding and allocation concealment might result in the overestimation of the effect of experimental treatments, causing bias. The quality of the included studies was not favorable, possibly decreasing the reliability of conclusions drawn in the present study.

### The effect of Er,Cr:YSGG laser on microleakage of restorations

With advances in dental materials, composite resin restorations have replaced amalgam restorations gradually. However, polymerization shrinkage, the major disadvantage of composite materials, can give rise to a marginal gap at the tooth–restoration interface, affecting the long-term success of restorations [10]. The phenomenon during which bacteria, liquids, molecules, or ions pass through the marginal gaps is known as microleakage and is often regarded as the most important factor resulting in secondary caries and pulpal infection [8, 21]. From this viewpoint, many suggestions have been made to reduce microleakage, such as the use of low-shrinkage resins and adequate preparation of the tooth [22]. Currently, the most common method for cavity preparation is the use of burs. However, the generated heat and pressure may cause pain and pulpal damage during cavity preparation if there is no adequate refrigeration, always posing a challenge for doctors and patients. For years, numerous new techniques have been developed as alternatives to traditional burs, of which the application of erbium family lasers has won broader and wider acceptance. Owing to its specific mechanism, Er,Cr:YSGG laser can cut enamel and dentin effectively, with less vibration, sharp noise, and pain for patients during cavity preparation. Also, adverse thermal effects on the pulp and surrounding tissues can be prevented effectively with the use of a water mist spray [23]. However, it should be noted that the parameters of lasers, such as repetition rate, air-water ratio, and mean power, are very critical as overheating can cause not only pulp damage but also undesirable morphological changes, such as cracks, carbonizations, etc., resulting in pain and irreversible damage. Some investigations have indicated that the Er,Cr:YSGG preparation improved the bonding process of adhesives and reduced microleakage [8, 24]. However, there is continuing controversy over the effect of Er,Cr:YSGG preparation on microleakage. Some studies found no difference between cavities prepared by bur and Er,Cr:YSGG laser [5, 15, 25], with some even reporting that Er,Cr:YSGG laser resulted in higher microleakage scores [24]. These reports have not been evaluated systematically to date.

Generally, microleakage tests can be conducted in vitro and in vivo; however, in vitro studies are more common owing to their precise and easy procedural steps [4]. For microleakage tests, several techniques have been widely used, including dye penetration, scanning electron microscopy, chemical tracers, air pressure, and neutron activation [5], with dye penetration being the most common technique for its ease of implementation, low cost, and safety [26]. Therefore, it is not surprising that all the studies included in our review were in vitro studies and used dye penetration for evaluation of microleakage.

As shown in our meta-analysis, there was no significant difference between Er,Cr:YSGG and bur preparations in terms of microleakage. Similarly, the same conclusion has been reached in a study by Rossi et al. With high absorbance in water and hydroxyapatite, the Er,Cr:YSGG laser beams can heat the water content of dental hard tissues, causing a micro-explosion of water particles [6]. The process of laser ablation produces an irregular, rough, and moist dental surface with exposed dentinal tubules, intact enamel rods, and no smear layer [5]. Considering these microscopic changes, some studies reported that Er,Cr:YSGG preparations might be more suitable for adhesion of restorative materials [8, 27]. However, some studies have indicated that the enamel melting, minimal cracking, and acid-resistant surfaces created by Er,Cr:YSGG irradiation during preparation might have adverse effects on the bonding process of adhesives, especially with ultra-mild self-etch adhesives [28–30].

Additionally, the ablation of dentin can fuse the collagen fibrils and reduce interfibrillar spaces, thus limiting the penetration of adhesives and resins [31]. Such unfavorable marginal sealing in Er,Cr:YSGG preparation has been observed by Geraldo-Martins et al. They measured the total length of the tooth–restoration interface and the interface infiltrated by dye to calculate the infiltration index [20]. The results indicated that Er,Cr:YSGG group had a higher microleakage index than the traditional bur group. The inconsistent conclusion drawn by Geraldo-Martins et al. might be explained by the fact that they used carious teeth as a study model and showed that laser could not remove the carious tissue completely, possibly affecting the marginal sealing of restorations. Under clinical conditions, bonding to dentin is more challenging because of its higher water and organic matter content. However, as shown in the present study, no significant difference was observed between the cavities prepared by lasers and those by burs in terms of microleakage rate, either on enamel or dentin margins.

It should be noted that there was significant heterogeneity among the included studies, and the conclusions reached in the present study should be interpreted cautiously. This heterogeneity can be explained by the variability in tooth type, cavity type, restoration materials, adhesives, and the irradiation parameters. For example, some studies used conventional methacrylate-based microhybrid composite resins for restorations, while some used low-shrinkage composite resin systems, which might have affected the results, accounting for the inconsistent conclusions [4, 9].

### The effect of acid etching on microleakage after laser preparation

Dental adhesives can be classified into two categories based on the way they react with the smear layer [32]. Etch-and-rinse adhesives need a prior acid etching procedure, while self-etch adhesives contain acidic functional monomers that can remove the smear layer selectively. In recent years, there has been a growing debate on the use of acid etching after laser preparation. Previous studies observed that prior acid etching improved the bond strength of self-etch and etch-and-rinse adhesives following laser preparation [33, 34]. However, some authors reported that acid etching did not affect the adhesive procedure in laser-prepared cavities, even weakening the bonding efficacy of self-etch adhesives [35, 36]. Based on these studies, acid etching might decrease the hydroxyapatite content of dental tissue, thereby weakening the chemical bonding of adhesives [12, 37]. In the present study, self-etch adhesives, in combination with prior acid etching, exhibited less microleakage than those without acid etching on enamel and dentin margins; however, the results were insignificant for etch-and-rinse adhesives. The following explanations might account for this result. According to many studies, a lack of water in dental tissue after laser preparation might affect the etching capacity of self-etching adhesives, thus limiting its penetration through the laser-modified layer. While additional acid etching can remove this layer, increase surface wettability, and benefit the hybrid layer formation, all of which can help reduce microleakage in laser-prepared cavities [12]. As reported by Obeidi et al., the etching time should be prolonged to 40 s, but not to 60 s, to improve the bond strength of self-etch adhesives in Er,Cr:YSGG-prepared teeth [32].

### Limitations of this study

This systematic review and meta-analysis had some limitations. Firstly, the effect of different restorative materials on microleakage was not fully considered. With inconsistent shrinkage rates, different resin-based systems might interfere with the results of further microleakage evaluation and cause bias.

Secondly, selecting an effective adhesive is also crucial for restorations. The applied adhesive systems varied widely among studies, including different self-etch and etch-and-rinse adhesives. Some studies showed that self-etch and etch-and-rinse adhesives had similar microleakage values in Er,Cr:YSGG laser preparations. However, the impact of different adhesives on microleakage should be further elucidated. Moreover, the differences in tooth characteristics, cavity type, and irradiation parameters might also affect microleakage. For example, although some studies showed that the morphology of primary teeth prepared by Er:YAG laser was similar to that of permanent teeth, the primary teeth often had a higher degree of mineralization, which might result in different reactions to laser beams.

Geraldo-Martins et al. indicated that it is essential to use carious teeth rather than healthy teeth as study models for the in vitro test of the microleakage. However, only one out of the 13 included studies used carious teeth to prepare cavities. Further studies are needed to verify the authenticity of healthy teeth as the research model in microleakage tests. The laser parameters, such as spot size, emission model, pulse duration, cooling rate, mean power, energy density, and focus, are vital for the application of lasers [23]. In addition, the chemical composition of the dental tissue influenced its efficacy. For example, the effect of laser on enamel was different from those observed in dentin. However, the laser parameters and the height of cavities varied markedly among the included studies, and some studies even lacked a detailed description of the information.

### Recommendations for future research

Considering the limitations mentioned above, the following suggestions are proposed for future research. Firstly, more RCTs should be conducted strictly according to the Cochrane’s criteria for the risk of bias. Secondly, researchers should report more data on the laser parameters applied in the study, and it is useful to explore the best parameters of Er,Cr:YSGG laser for cavity preparation. Thirdly, further investigations should focus on the long-term effects of laser preparation on restorations. Lastly, it is necessary to demonstrate whether it is possible to use sound teeth as study models to prepare cavities and test the microleakage of restorations.

## Conclusions

Considering the data obtained, Er,Cr:YSGG laser application resulted in microleakage similar to that with a traditional bur, irrespective of the tooth type, cavity type, and restorative materials. Adjunctive use of acid etching is recommended with self-etching adhesives after Er,Cr:YSGG preparations. However, given the limitations of the current study, further investigations are urgently needed to reach a more valid conclusion.

## Data Availability

All data generated or analyzed during this study are included in this published article and its supplementary information files.

## Abbreviations

RCTs: Randomized controlled trials
Er,Cr:YSGG: Erbium, Chromium Yttrium Scandium Gallium Garnet
FDA: Food and Drug Administration

## References

[1] Valério RA, Borsatto MC, Serra MC (2015). Caries removal in deciduous teeth using an Er:YAG laser: a randomized split-mouth clinical trial. Clin Oral Investig.

[2] Chen Y, Liu C, Chen X (2019). Clinical evidence of photobiomodulation therapy (PBMT) on implant stability and success: a systematic review and meta-analysis. BMC Oral Health.

[3] Hibst R (2002). Lasers for caries removal and cavity preparation: state of the art and future directions. J Oral Laser Appl.

[4] Beheshteh M, Mohammad A, Fateme J (2017). Comparison of marginal microleakage of flowable composite restorations in primary canine teeth prepared with high-speed diamond bur, Er:YAG laser and Er,Cr:YSGG laser. Laser Ther.

[5] Subramaniam P, Pandey A, Subramaniam P (2016). Assessment of microleakage of a composite resin restoration in primary teeth following class III cavity preparation using Er, Cr:YSGG laser: an in vitro study. J Lasers Med Sci.

[6] Ergin E, FD OZ, Gurgan S (2018). Comparison of Er,Cr:YSGG Laser Handpieces for Class II Preparation and Microleakage of Silorane- or Methacrylate-Based Composite Restorations. Photomed Laser Surg.

[7] Shahabi S, Ebrahimpour L, Walsh LJ (2010). Microleakage of composite resin restorations in cervical cavities prepared by Er,Cr:YSGG laser radiation. Aust Dent J.

[8] Fattah T, Kazemi H, Fekrazad R (2013). Er,Cr:YSGG laser influence on microleakage of class V composite resin restorations. Lasers Med Sci.

[9] Shafiei F, Memarpour M (2015). Effect of acid etching on long-term microleakage of Nano Ionomer restorations in Burand laser-prepared cavities in primary teeth. J Dent Child.

[10] Bahrololoomi Z, Heydari E (2014). Assessment of tooth preparation via Er:YAG laser and bur on microleakage of dentin adhesives. J Dent.

[11] Borsatto MC, Corona SA, Chinelatti MA (2006). Comparison of marginal microleakage of flowable composite restorations in primary molars prepared by high-speed carbide bur, Er:YAG laser, and air abrasion. J Dentr Child.

[12] Shafiei F, Memarpour M, Fekrazad R (2014). Sealing of silorane-based composite in laser-prepared primary teeth: effect of acid etching. Pediatr Dent.

[13] Liberati A, Altman DG, Tetzlaff J (2009). The PRISMA statement for reporting systematic reviews and meta-analyses of studies that evaluate healthcare interventions: explanation and elaboration. BMJ.

[14] Landis JR, Koch GG (1977). The measurement of observer agreement for categorical data. Biometrics.

[15] Yazici AR, Yıldırım Z, Antonson SA (2012). Comparison of the Er,Cr:YSGG laser with a chemical vapour deposition bur and conventional techniques for cavity preparation: a microleakage study. Lasers Med Sci.

[16] Gutknecht N, Apel C, Schafer C (2001). Microleakage of composite fillings in Er,Cr:YSGG laser-prepared class II cavities. Lasers Surg Med.

[17] Trelles K, Arnabat J, Espana-Tost T (2012). Microleakage in Class V cavities with self-etching adhesive system and conventional rotatory or laser Er,Cr:YSGG. Laser Ther.

[18] Marotti J, Geraldo-Martins VR, Bello-Silva MS (2010). Influence of etching with erbium, chromium:yttrium-scandium-gallium-garnet laser on microleakage of class V restoration. Lasers Med Sci.

[19] Rossi RR, Aranha AC, Eduardo CP (2008). Microleakage of glass ionomer restoration in cavities prepared by Er,Cr:YSGG laser irradiation in primary teeth. J Dent Child.

[20] Geraldo-Martins V, Thome T, Mayer M (2013). The use of bur and laser for root caries treatment: a comparative study. Oper Dent.

[21] Alani AH, Toh CG (1997). Detection of microleakage around dental restorations: a review. Oper Dent.

[22] Oskoee PA, Kimyai S, Ebrahimi Chaharom ME (2012). Cervical margin integrity of class II resin composite restorations in laser- and bur-prepared cavities using three different adhesive systems. Oper Dent.

[23] Gurgan S, Alpaslan T, Kiremitci A (2009). Effect of different adhesive systems and laser treatment on the shear bond strength of bleached enamel. J Dent.

[24] Moldes VL, Capp CI, Navarro RS (2009). In vitro microleakage of composite restorations prepared by Er:YAG/Er,Cr:YSGG lasers and conventional drills associated with two adhesive systems. J Adhes Dent.

[25] Hossain M, Yamada Y, Nakamura Y (2003). A study on surface roughness and microleakage test in cavities prepared by Er:YAG laser irradiation and etched bur cavities. Lasers Med Sci.

[26] Taylor MJ, Lynch E (1992). Microleakage. J Dent.

[27] Esteves-Oliveira M, Zezell DM, Apel C (2007). Bond strength of self-etching primer to bur cut, Er,Cr:YSGG, and Er:YAG lased dental surfaces. Photomed Laser Surg.

[28] Cardoso MV, Coutinho E, Ermis RB (2008). Influence of Er,Cr:YSGG laser treatment on the microtensile bond strength of adhesives to dentin. J Adhes Dent.

[29] Mine A, Yoshida Y, Suzuki K (2006). Spectroscopic characterization of enamel surfaces irradiated with Er:YAG laser. Dent Mater J.

[30] Souza-Zaroni WC, Chinelatti MA, Delfini CS (2008). Adhesion of a self-etching system to dental substrate prepared by Er:YAG laser or air abrasion. J Biomed Mater Res B Appl Biomater.

[31] Ceballos L, Osorio R, Toledano M (2001). Microleakage of composite restorations after acid or Er-YAG laser cavity treatments. Dent Mater.

[32] Perdigão J (2007). New developments in dental adhesion. Dent Clin N Am.

[33] Obeidi A, Liu PR, Ramp LC (2010). Acid-etch interval and shear bond strength of Er,Cr:YSGG laser-prepared enamel and dentin. Lasers Med Sci.

[34] Eguro T, Maeda T, Otsuki M (2002). Adhesion of Er:YAG laser-irradiated dentin and composite resins: application of various treatments on irradiated surface. Lasers Surg Med.

[35] Van-Meerbeek B, Yoshihara K, Yoshida Y (2011). State of the art of self-etch adhesives. Dentl Mater.

[36] Rengo C, Goracci C, Juloski J (2012). Influence of phosphoric acid etching on microleakage of a self-etch adhesive and a self-adhering composite. Aust Dent J.

[37] Duarte SJR, Phark JH, Varjao FM (2009). Nanoleakage, ultramorphological characteristics, and microtensile bond strengths of a new low-shrinkage composite to dentin after artificial aging. Dent Mater.

